# Evaluation of NPP1 as a Novel Biomarker of Coronary Artery Disease: A Pilot Study in Human Beings

**DOI:** 10.15171/apb.2018.057

**Published:** 2018-08-29

**Authors:** Amir Hooshang Mohammadpour, Saeed Nazemi, Fatemeh Mashhadi, Atefeh Rezapour, Mohammad Afshar, Sepideh Afzalnia, Afsaneh Mohammadi, Hamid Reza Mashreghi Moghadam, Maryam Moradian, Seyed Mohammad Hasan Moallem, Saeed Falahaty, Azadeh Zayerzadeh, Sepideh Elyasi

**Affiliations:** ^1^Pharmaceutical Research Center, Mashhad University of Medical Sciences, Mashhad, Iran.; ^2^Department of Clinical Pharmacy, School of Pharmacy, Mashhad University of Medical Sciences, Mashhad, Iran.; ^3^Research and Education Department, Razavi Hospital, Mashhad, Iran.; ^4^Department of Anatomy, Birjand University of Medical Sciences, Birjand, Iran.; ^5^Medical Toxicology Research Center, Mashhad University of Medical Sciences, Mashhad, Iran.; ^6^Birjand Cardiovascular Disease Research Center; Department of Cardiology, Birjand University of Medical Sciences, Birjand, Iran.; ^7^Department of Pediatric Cardiology, Rajaie Cardiovascular Medical and Research Center, Tehran, Iran.; ^8^School of Medicine, Mashhad University of Medical Sciences, Mashhad, Iran.

**Keywords:** Coronary Artery Calcification, ENPP1, Biomarker, Inorganic pyrophosphate, Glycoprotein 1- nucleotides

## Abstract

***Purpose:*** Coronary artery calcification (CAC) is utilized as an important tool for global risk assessment of cardiovascular events in individuals with intermediate risk. Ecto phosphodiesterase/nucleotide phosphohydrolase-1(ENPP1) converts extracellular nucleotides into inorganic pyrophosphate and it is a key regulator of tissue calcification that adjusts calcification in tissues like vascular smooth muscle cells. The main purpose of this clinical study was to find out the correlation between ENPP1 serum concentration and CAC in human for the first time.

***Methods:*** In this study 83 patients (16 diabetic patients and 67 non-diabetic patients) with coronary artery disease who fulfilled inclusion and exclusion criteria, entered the study. For all patients a questionnaire consisting demographic data and traditional cardiovascular risk factors were completed. Computed tomography (CT)-Angiography was carried out to determine coronary artery calcium score and enzyme-linked immunosorbent assay (ELISA) method was used for measuring ENPP1 serum concentrations.

***Results:*** There was a reverse significant correlation between ENPP1 serum concentration and total CAC score and also CAC of right coronary artery (RCA) (P<0.05) in non-diabetic patients.

***Conclusion:*** On the basis of our results, ENPP1 serum concentration may be a suitable biomarker for coronary artery disease at least in non-diabetic patients. However, more studies with higher sample size are necessary for its confirmation.

## Introduction

Vascular calcification is a life threatening complication of cardiovascular disease and an independent risk factor for high morbidity and mortality.^1^ It is an inevitable process particularly in the advanced stages of atherosclerosis which can cause the plaque rupture. Coronary artery calcification (CAC) is a surrogate marker for subclinical atherosclerosis and recently determined as strong predictor that comforts the prediction of future cardiovascular events particularly in intermediate risk subjects. It is determined by electron beam-computed tomography (EBCT).^2^ Increased coronary artery calcium score (CACS) correlates with the risk of cardiovascular disease.^3^

Recent studies have provided impetus to shift from cellular interaction based calcification models to models emphasizing on the important role of extracellular matrix in calcification. Adenosine triphosphate (ATP) and other nucleotides and nucleosides play different biochemical roles depending on differential tissue expression, cell distribution, and substrate availability and their presence in either the intracellular or extracellular compartment. Ecto-nucleotidases classified to four families including ecto-nucleotide pyrophosphate / phosphor - diesterase (ENPP) family. ENPP1 is a member of ENPP family that expresses in different tissues including cartilage, kidney, heart, parathyroid and skeletal muscle, and to a greater extent in vascular smooth muscle cells (VSMCs), osteoblasts and chondrocytes.^4-6^ NPP1 is known to play vital roles in calcium/phosphate regulation, and repression of soft tissue mineralization, and maintaining skeletal structure and function. NPP1 hydrolyses ATP to produce either inorganic pyrophosphate (PP_i_) plus adenosine monophosphate (AMP) or inorganic phosphate (P_i_) plus adenosine diphosphate (ADP) in a two stage process via either ADP or a phosphate bound intermediate.^7-9^ PP_i_ is a central regulator of calcification in the extracellular matrix. In extracellular, PP_i_ draw ups gene expression and cellular differentiation, which have main physiologic effects on chondrogenesis and expression of osteopontin. PP_i_ strongly inhibits the nucleation and advancement of hydroxyapatite (HA) and other basic calcium phosphate crystals.^7,10,11^ Therefore, through generating PPi, NPP1 is a key regulator of tissue calcification and bone development and can be effective in prevention of pathologic tissue calcification.

According to this, we evaluated the ENPP1 as a diagnostic biomarker in human to determine the extent of coronary artery calcification.

## Materials and Methods

### Patients

Eighty-three patients, who aged higher than 40 years old with diagnosis of coronary artery disease by angiography which was performed by the cardiologist, were enrolled in this study between November 2015 and March 2016. This test is the best way to detect coronary artery disease (CAD) in the arteries, over 51% of which are blocked by atherosclerotic plaques and useful in detecting the vessels responsible for advanced CAD. However, it does not provide information about the artery wall and atherosclerosis may not be diagnosed that has not yet captured the duct.^12^ patients with >50% coronary stenosis of at least one artery were considered as CAD+ and included in study. Patients were recruited from Cardiology ward of Razavi Hospital, Mashhad, Iran. This study was accepted by ethics committee of Mashhad University of Medical Sciences (code: 931459). All patients signed the consent form prior to entry in the study. All patients signed the consent form prior to entry in the study.

Patients with calcium and phosphor metabolic disorder or receiving medications which are effective on calcium and/or phosphate and immunosuppressant or antioxidant medications, intake of folic acid and methotrexate, malignancies, heart failure, hypo or hyper parathyroidisim, renal insufficiency, history of osteoarticular disorders and chronic inflammatory diseases, and acute infection during the study were excluded from the study. A questionnaire containing demographic data, laboratory data, drug and medical and familial history of cardiovascular risk factors was completed for all patients.

### Determination of ENPP1 serum concentration and CAC

Twenty milliliter of whole blood was collected from patients and centrifuged at 2500 rpm for 10 min. Two milliliter serum were isolated and divided into 4 micro sets of 0.5 ml. The serum was stored at -70 °C until required for analysis. Routine biochemical measurements such as plasma glucose, total cholesterol (TC), triglycerides, low density lipoprotein Cholesterol (LDL-c), high-density lipoprotein cholesterol (HDL-c), and serum calcium and phosphorus level were carried out by routine laboratory methods. Serum level of soluble ENPP1 was measured with an enzyme-linked Immunosorbant assay (ELISA) -kit (Zellbio, Germany); each assay was calibrated using ENPP1 standard curve following the manufacturer^‘^s instructions. Coronary Artery Calcification score of left main coronary artery (LMCA), left anterior descending (LAD) and circumflex (CX) and right coronary artery (RCA) was determined by high resolution B mode ultrasonography in radiology department of Razavi Hospital. CAC measurement is now considered a potentially useful test for improving coronary risk assessment in selected intermediate-risk asymptomatic patients in whom high CAC scores signify increased cardiovascular risk beyond that predicted by conventional cardiovascular risk factors alone. Agatston score is a semi-automated tool to calculate a score based on the extent of coronary artery calcification detected by an unenhanced low-dose CT scan which is routinely performed in patients undergoing cardiac CT. Due to an extensive body of search, it allows for an early risk stratification as patients with a high Agatston score (>160) have an increased risk for a major adverse cardiac event. Although it does not allow for the assessment of soft non-calcified plaques, it has shown a good correlation with contrast enhanced CT coronary angiography.^13,14^

However, it should be defined that when the pretest probability of coronary artery disease is low (eg, asymptomatic screening setting), a CAC score of zero is associated with low risk of coronary artery disease and low risk of near-term coronary events but in older asymptomatic patients with risk factors, CAC=0 is associated with a moderate increased risk of events and in patients with clinical signs and symptoms associated with an intermediate-to-high risk of coronary disease, CAC=0 is often associated with myocardial ischemia on provocative testing and with a high risk of near-term coronary events.^15^

### Statistical Analysis

Data recruited from the standard forms were gathered and then analyzed with SPSS version 16.0 (Systat Software, Inc., Chicago, IL). For descriptive assessment, mean ± standard deviations of continuous variables were provided. For nominal variables, number and percentages were reported. Correlation between Serum Concentration of ENPP1 with CAC was analyzed using spearman correlation test. Chi-squared test and t test were applied for continuous and nominal data, where appropriate. Results were considered significant at p<0.05.

## Results

### Characteristics of the study Population

The study population consists of 83 patients, male (77%) and female (23%). The mean age of population was 56.80±10.73 years. Patients’ characteristics, laboratory tests including biochemical parameters, and traditional cardiovascular risk factors and mean ENPP1 serum level are summarized in Table 1. Moreover, these factors are defined in diabetic and non-diabetic patients separately and female/male ratios, positive family history of CAD and hypertension prevalence were significantly different between these two groups.

**Table 1:** patients characteristic, laboratory data, traditional cardiovascular risk factors and mean ENPP1 serum level of patients

**Patients characteristic**	**All patients (n=83) (Mean±SD)**	**Diabetic patients (n=16) (Mean±SD)**	**Non-diabetic patients (n=67) (Mean±SD)**	**P value**
Age (year)	57.13±10.7	56.4±10.52	57.74±10.65	0.95^1^
BMI (kg/m^2^)	28.36±4.78	28.46±3.8	27.99±5.46	0.43^1^
Female/male ratio	0.29	0.54	0.32	0.005*^2^
Laboratory tests	**Mean±SD**			
HDL-C (mg/dl)	41.92±9.97	40.76±9.14	45.24±15.68	0.66^1^
LDL-C (mg/dl)	90.81±29.14	95.88±34.56	88.69±28.04	0.06^1^
Total cholesterol (mg/dl)	163.30±33.32	165.76±38.28	164±33.81	0.09^1^
FBS (mg/dl)	104.56±24.00	141.2±44.05	97.57±14.71	0.005*^1^
Traditional risk factors	**Frequency (%)**			
Hypertension (%)	45.88	60	45.34	0.005*^2^
Dyslipidemia (%)	63.52	65	61.62	0.63^2^
Positive family history (%)	51.76	47.62	62.79	0.005*^2^
Diabetes (%)	20.58	100	0	-
Current Smoking (%)	35.29	33.33	23.25	0.66^2^
Concentration of ENPP1 (pg/mL)	106.4214±78.54876	132.62±100.5	100.26±71.97	0.14^1^

BMI: Body Mass Index, HDL-C: High Density Lipoprotein-Cholesterol, LDL-C: Low Density Lipoprotein-Cholesterol, FBS: Fast Blood Sugar, ENPP1: Ecto-nucleotide pyrophosphate/phosphor-di-esterase ^1^ independent sample T test ^2^chi square * P value<0.05 is considered significant

### Correlation between ENPP1 serum level and Coronary Artery Calcification agatson score 

There was a reversed significant correlation between ENPP1 serum level and total coronary artery calcification score and CAC score of RCA in non-diabetic patients (P<0.05) but, there was no significant correlation between ENPP1 serum level and CAC score of LMCA, LAD and CX.(p>0.05) (Table 2)

**Table 2:** Correlation between ENPP1 serum concentration with LAD, RCA, LMCA, and CX coronary artery calcification score

**Coronary artery Calcium score**	**Mean ±SD**	**P value Spearman Correlation Test**	**Correlation coefficient**
Total calcification of coronary vessels (agatson score)	357.29±590.81	0.004	-0.121
Calcification in coronary LAD(agatson score)	184.60±304.46	0.345	0.044
Calcification in coronary RCA(agatson score)	63.37±101.86	0.00	-0.22
Calcification in coronary CX (agatson score)	44.86±99.00	0.416	-0.264
Calcification in coronary LMCA (agatson score)	34.11±116.00	0.494	-0.107

CAC: Coronary Artery Calcification, LAD: Left Anterior Descending, RCA: Right Coronary Artery LMCA: Left Main Coronary Artery, CX: Circumflex

Moreover, based on Rumberger method we divided patients based on their total CAC to three groups; mild (CAC lower than 25^th^ of range), moderate (between 25^th^ and 75^th^ of range) and severe (≥75^th^ of CAC range). Fifty-four percent of the patients were in mild group and 44.4% and 1.6% were in moderate and severe groups, respectively. There was no significant difference between ENPP1 serum level of these groups (P>0.05).

## Discussion

In this study, the correlation of the ENPP1 serum level with CAC was evaluated for the first time in human. As mentioned in results, there was a significant reversed correlation between ENPP1 serum level and total and RCA CAC score in non-diabetic patients (P<0.05) but no significant correlation with CAC score of LAD, LM and CX (P ≥0.05). Several In vitro and In vivo studies have been conducted on the relationship between coronary artery calcification and serum ENPP1 level previously but they were only limited to the examination of genetic disorders and gene mutations of ENPP1 in infants and evaluation of the relationship between serum levels of ENPP1 and calcification of atherosclerotic plaques in diabetic patients.

Based on previous studies, it is entirely apparent that the calcification is suppress by ENPP1 by means of PP_i_ that it is a potent inhibitor of hydroxyl apatite (HA) crystal formation in mineralized competent of tissues.

NPPs can convert AMP into adenosine and P_i_, although conflicting reports suggest that AMP competitively inhibits NPP activity. PPi is hydrolyzed by tissue-nonspecific alkaline phosphatase (TNAP) into inorganic phosphate (Pi), which co-crystallizes with calcium into HA and thereby promotes bone formation. Thus, PPi has a dual role as it can both suppress and promote HA crystal deposition, depending on the expression ratio and catalytic activities of NPP1 and TNAP. The distorted balance of Pi/PPi ultimately leads to pathological calcification.^16-19^ In another study, NPP1-deficient mice showed reduced levels of extracellular PPi, causing pathological calcification of cartilage and soft tissues, such as arterial smooth muscle walls and also abnormal bone development.^16,17^ So, according to the previous studies, it is clear that NPP1 and PP_i_ physiologically work to prevent calcification of arteries and certain other soft tissues.^20,21^ This study was the first clinical study that evaluated the relationship between serum concentrations of ENPP1 and coronary artery calcification in patients with chronic heart ischemia. According to the results mentioned above, there was a significant reversed relationship between the coronary artery calcification and this biomarker in non-diabetic patients.

A study was conducted in 2010 by Jeong et al. which assessed the relationship between coronary artery calcification and ENPP1 gene expression levels in 140 diabetic patients. None of patients had history of cardiovascular disease and no relationship was found between coronary artery calcification and ENPP1 gene expression levels.^22^ In present study 16 diabetic patients and 67 non-diabetic patients were included and no relationship was found between total coronary artery calcification score and serum concentrations of ENPP1. However, after exclusion of diabetic patients a significant relationship was observed (P = 0.004). Nowadays type 2 diabetes prevalence is increasing steadily all over the world. One of the criteria for type 2 diabetes, is insulin resistance in different body tissues such as skeletal muscle, liver and fat tissues that occurs due to impaired peripheral receptor signaling of insulin. In several studies it has been observed that ENPP1, which is connected directly to the insulin receptor α, impairs receptor function and then reduces the signaling cascade, in a wide variety of tissues such as skeletal muscle and liver.^23^ In some other studies has been observed that the expression level of ENPP1 in patients with insulin resistance has been increased and also it is mentioned that regulatory increased ENPP1level in rat liver, induces insulin resistance and glucose tolerance.^24-26^

In a multicenter clinical study conducted in Italy and America in 2005, it is observed that increased expression of ENPP1 is associated with a higher prevalence of diabetes and myocardial infarction.^27^ So, in this research, we excluded patients with type 2 diabetes and it was found that the level of serum ENPP1 is affected by type 2 diabetes and there is a significant negative relationship between the ENPP1 serum levels and the total calcification score of coronary arteries in non-diabetic patients with cardiovascular disease.

This significant negative correlation was also found by CAC of RCA but not the other coronary arteries. It may be due to non-uniform distribution of other vessels’ calcium scores resulting from small sample size. So, limited number of patients included in this study is the major limitation of this study.

## Conclusion

In this study, the correlation of the ENPP1 serum level with CAC was clinically evaluated for the first time in patients with coronary artery disease. There was a reverse significant correlation between ENPP1 serum level and total CAC and CAC of RCA in non-diabetic patients (P<0.05), but there was no significant correlation between ENPP1 serum level patients and LAD, LM and CX (P >0.05). Further studies are recommended in this field with higher sample size.

## Acknowledgments

This study is part of a research thesis for a Pharm.D. degree at Mashhad University of Medical Sciences.

The authors are thankful for the funding of this study by the Research Council of Mashhad University of Medical Sciences.

## Ethical Issues

The study protocol was approved by the ethical committee of Mashhad University of Medical Sciences.

## Conflict of Interest

The authors have no conflicts of interest.
